# A neural network for glomerulus classification based on histological images of kidney biopsy

**DOI:** 10.1186/s12911-021-01650-3

**Published:** 2021-11-01

**Authors:** Giacomo Donato Cascarano, Francesco Saverio Debitonto, Ruggero Lemma, Antonio Brunetti, Domenico Buongiorno, Irio De Feudis, Andrea Guerriero, Umberto Venere, Silvia Matino, Maria Teresa Rocchetti, Michele Rossini, Francesco Pesce, Loreto Gesualdo, Vitoantonio Bevilacqua

**Affiliations:** 1 Department of Electrical and Information Engineering (DEI), Polytechnic University of Bari, Bary, Italy; 2 Apulian Bioengineering s.r.l., Modugno, BA Italy; 3 Department of Emergency and Organ Transplantation, Nephrology Unit University of Bari Aldo Moro, Bari, Italy

**Keywords:** CKD, Kidney, Glomerulus classification, Morphological features, Texture features, ANN

## Abstract

**Background:**

Computer-aided diagnosis (CAD) systems based on medical images could support physicians in the decision-making process. During the last decades, researchers have proposed CAD systems in several medical domains achieving promising results.

CAD systems play an important role in digital pathology supporting pathologists in analyzing biopsy slides by means of standardized and objective workflows. In the proposed work, we designed and tested a novel CAD system module based on image processing techniques and machine learning, whose objective was to classify the condition affecting renal corpuscles (glomeruli) between sclerotic and non-sclerotic. Such discrimination is useful for the biopsy slides evaluation performed by pathologists.

**Results:**

We collected 26 digital slides taken from the kidneys of 19 donors with Periodic Acid-Schiff staining. Expert pathologists have conducted the slides preparation, digital acquisition and glomeruli annotations. Before setting the classifiers, we evaluated several feature extraction techniques from the annotated regions. Then, a feature reduction procedure followed by a shallow artificial neural network allowed discriminating between the glomeruli classes.

We evaluated the workflow considering an independent dataset (i.e., processing images not used in the training procedure). Ten independent runs of the training algorithm, and evaluation, allowed achieving MCC and Accuracy of 0.95 (± 0.01) and 0.99 (standard deviation < 0.00), respectively. We also obtained good precision (0.9844 ± 0.0111) and recall (0.9310 ± 0.0153).

**Conclusions:**

Results on the test set confirm that the proposed workflow is consistent and reliable for the investigated domain, and it can support the clinical practice of discriminating the two classes of glomeruli. Analyses on misclassifications show that the involved images are usually affected by staining artefacts or present partial sections due to slice preparation and staining processes. In clinical practice, however, pathologists discard images showing such artefacts.

## Background

Chronic Kidney Disease (CKD) is a pathological condition characterized by a functional degeneration of the kidney. CKD is the 12^th^ cause of death, with up to 1.1 millions cases worldwide; the increased mortality related to CKD of the last years makes it one of the fastest rising causes of death, alongside diabetes and dementia [1, 2]. Kidney transplantation is the best renal replacement therapy as revealed to be more effective than dialysis treatment in terms of long-term mortality risk and, at the same time, has a reduced impact on the public health system [3, 4].

Liyanage et al. estimated that 2.6 million people, in the face of 4.9 million patients, received renal replacement therapy worldwide in 2010, suggesting that at least 2.3 million people might have died prematurely because appropriate therapy could not be accessed [5].

Due to the increasing necessity of kidney transplants [6], different studies tried to widen the criteria for accepting kidneys for being transplanted, which are generally excluded based on the donor’s age and other characteristics related both to the quality and dimension of kidneys [7, 8].

Moore et al. performed a comparison between dual kidney transplantation from Expanded Criteria Donors (ECDs) and single kidney transplantation from concurrent ECDs and standard criteria donors. The authors assessed that the use of dual kidney transplantation from marginal donors is a viable option and that renal function can be achieved, provided that both kidneys are transplanted into a single recipient [9].

Remuzzi et al. proposed a technique to assess the kidney condition by evaluating histological biopsies [10]. The evaluation criterion, known as the Karpinski score, considers the evolution (in percentage) of a pathological condition of four main functional areas: glomerulosclerosis, tubular atrophy, interstitial fibrosis and arterial sclerosis. This score ranges from 0 to 12, and the higher the number, the worse is the kidneys’ condition [10–12]. Kidneys with a Karpinski score from 0 to 3 and from 4 to 6 are considered suitable for single and dual transplant, respectively.

To assess the Karpinski score, pathologists perform the visual evaluation of the histopathological Whole-Slide Images (WSIs). This process is usually time-consuming, prone to error and also subjective.

To overcome these drawbacks, the development of Computer-Aided Diagnosis (CAD) systems based on histopathological tissue image analysis for supporting the computation of the score is a valuable headway.

Recent literature works show the application of image processing and machine learning techniques to analyze kidney histopathological WSIs for glomeruli detection and classification. Image processing approaches aim to extract meaningful features, e.g., those based on shape and texture analysis; then, machine learning algorithms, such as shallow or deep Artificial Neural Networks (ANNs), make decisions based on extracted features.

Simon et al., for example, proposed a texture-based features set as a simple but effective automatic method for glomeruli localization [13]. The authors applied the algorithm on renal tissue sections and biopsies of large histopathological WSIs. The features extracted from an adaptation of the Local Binary Pattern (LBP) algorithm were used to train a Support Vector Machine (SVM) model. The authors reported high precision (> 90%) and reasonable recall (> 70%) as results.

To perform a comprehensive detection of glomeruli in images of whole kidney sections, Kato et al. proposed a new descriptor called Segmental HOG (Histogram of Oriented Gradients) [14]. The authors claimed the robustness of the solution and high-quality segmentation outputs; furthermore, the authors compared Segmental HOG with Rectangular HOG showing that the first approach reached significant improvements in detection performance.

Several authors, instead, focused on the analysis of glomeruli’s shape and colour. Kotyk et al. proposed a novel solution to face the wide intensity variation and the inconsistency in terms of shape and size of the glomeruli in the renal corpuscle. The proposed approach, based on Particles Analyzer technique, allowed the detection of the renal corpuscle and the following measurement of glomerulus diameter and Bowman’s space width. The authors assess that the approach was robust to glomeruli deformations even with glomerular hypertrophy [15]. An analysis of the effects of significant diversity of colour and tissue shape on whole slide images was performed by Zhao et al. [16]. The authors focused on the extraction of Bowman’s capsule width to design an automated glomerulus extraction framework from the micrograph of the entire renal tissue. The system was tested on non-human primates renal tissues with Haematoxylin and Eosin (HE) staining.

Bukowy et al. proposed a different analysis workflow. In [17], the authors developed a convolutional neural network to detect glomeruli in trichrome-stained kidney sections. The procedure was tested on rat kidneys and the reported results, regarding the classification of healthy and damaged glomeruli, show average precision and recall of 96.94% and 96.79%, respectively.

In a previous work by Bevilacqua et al., a CAD system for segmentation and discrimination of blood vessels versus tubules from biopsies in the kidney tissue has been designed and tested [18]. Histological images with Periodic Acid–Schiff (PAS) staining have been used to segment Regions of Interest (ROIs) and extract Haralick features allowing a subsequent classification procedure using algorithms based on ANNs. Test results determined that the supervised ANN approach was consistent, allowing obtaining good classification performance.

This work focuses on the automatic evaluation of kidney biopsies, dealing with a specific pathological condition considered by the Karpinski score: glomerulosclerosis, i.e. the ratio between sclerosed glomeruli and the overall number of glomeruli. To do this, the detection and discrimination of the sclerotic condition affecting the glomeruli from those non-sclerotic are crucial. As already reported in works from the state-of-the-art, this is a challenging task due to the glomeruli wide intensity variations and inconsistencies in shape and size.

A combination of different feature extraction algorithms has been designed and evaluated for discriminating the condition of glomeruli. The reported literature shows specific and unique image processing algorithms applied on different types of staining and non-human WSIs. The set of features proposed in this work, instead, comes from a collection of two wide-used, well-known and general-purpose features extractor algorithms families, i.e. morphological and texture features. These feature families are also included in some of the algorithms proposed in literature, but in this work they were extracted from human WSIs with PAS staining. In addition, the classification pipeline, detailed in Methods, includes also procedures for features reduction allowing the design of a shallow Artificial Neural Network. The overall workflow proposed in this work, and the integration with the procedure presented in [18], will allow us to build-up a complete CAD system for the analysis of histopathological WSIs.

## Results

The results obtained by evaluating the proposed classification workflow on the test set are reported. In particular, results refer to the performance obtained considering the reduced set of features classified by using the cross-validated shallow ANN. As reported in Table 1, the test set was constituted by 579 glomeruli images: 87 sclerotics, 492 non-sclerotics.

**Table 1:** Dataset configuration

Dataset	Sclerotic glomeruli	Non-sclerotic glomeruli	Total
Train set	341	1852	2193
Test set	87	492	579
Total	428	2344	2772

To evaluate the workflow stability, 10 runs of the entire process were performed. The achieved results are summarized in Table 2. In particular, the results are reported in terms of mean and standard deviation of several metrics, i.e. Accuracy (Eq. 1), Precision (Eq. 2), Recall (Eq. 3) and Matthews Correlation Coefficient (Eq. 4) [19], evaluated according to the confusion matrix reported in Table 3.

**Table 2:** Metrics comparison of 10 network initializations

	Mean ± std
Accuracy	0.9874 ± 0.0018
Precision	0.9844 ± 0.0111
Recall	0.9310 ± 0.0153
MCC	0.9501 ± 0.0074

**Table 3:** Confusion Matrix for metrics computation

		True condition	
		Positive (sclerotic)	Negative (non-sclerotic)
*Predicted condition*	Positive (sclerotic)	True positive (TP)	False positive (FP)
	Negative (non-sclerotic)	False negative (FN)	True negative (TN)

Among the iterations, the best results are reported in Table 4, whereas the corresponding confusion matrix is reported in Table 5.

1 $$Accuracy=\frac{{TP + TN}}{{TP + TN + FP + FN}}$$

2 $$Precision = \frac{{TP}}{{TP + FP}}$$

3 $$Recall = \frac{{TP}}{{TP + FN}}$$

4 $$MCC = \frac{{TP{\text{*}}TN - FP{\text{*}}FN}}{{\sqrt[2]{{\left( {TP + FP} \right){\text{*}}\left( {TP + FN} \right){\text{*}}\left( {TN + FP} \right){\text{*}}\left( {TN + FN} \right)}}}}$$

The implemented workflow allows the classification of sclerotic and non-sclerotic glomeruli with good performances (mean MCC = 0.95 and mean Accuracy = 0.99) and low variability (MCC std = 0.01 and Accuracy std < 0.00) (see Table 2). Precision and Recall are equal to 0.98 and 0.93, respectively, thus showing that the proposed system achieves a better performance in the non-sclerotic evaluation (all the non-sclerotic glomeruli were detected in the best case).

**Table 4:** Metrics comparison of 10 network initializations

Metric	Performance
Accuracy	0.9914
Precision	1.000
Recall	0.9425
MCC	0.9659

**Table 5:** Confusion matrix of the best model

		True condition	
		Positive (sclerotic)	Negative (non-sclerotic)
*Predicted condition*	Positive (sclerotic)	True positive (82)	False positive (0)
	Negative (non-sclerotic)	False negative (5)	True negative (492)

## Discussion

Evaluating the proposed approach on an independent test set, the classification workflow achieved a mean MCC and Accuracy of 0.95 and 0.99, respectively, and low variability over 10 independent iterations (MCC std = 0.01 and Accuracy std < 0.00). Good precision and recall were also obtained (Precision: 0.9844 ± 0.0111, Recall: 0.9310 ± 0.0153). The proposed approach thus leads to an improvement of the classification performance if compared to the reported literature [13, 17].

While implementing and evaluating the reported workflow, we faced and tested the common data unbalancing problem, that has been solved by using MCC as performance comparison coefficient and ROC curve for selecting the optimal classification threshold. The reported results suggest that the proposed workflow set-up is reliable for the investigated domain, supporting the clinical practice of discriminating the two classes of glomeruli.

**Fig. 1:**
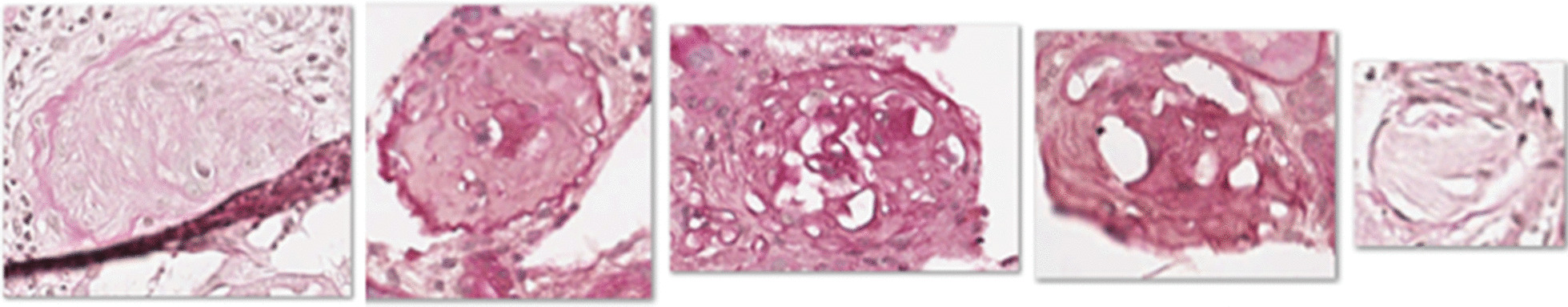
False Negative misclassified by the best model

Analyzing misclassified glomeruli, we found also that the input images corresponding to the misclassified samples showed staining artefacts or partial parts (mostly on the edges); common examples are mentioned in Fig. 1. In the clinical practice, however, pathologists discard such images which could also be excluded in the proposed workflow by designing strategies for detecting in advance images affected by such problems.

## Conclusions

In the presented work, we proposed an entire workflow for the classification of sclerotic and non-sclerotic glomeruli. Several feature extraction algorithms were examined and evaluated, with two feature typologies being chosen: morphological and texture features. We collected 150 features: 2 morphological features and 148 texture ones that have been computed using the mrcLBP and Haralick algorithms. The number of features was then reduced to 95 using the PCA. A cross-validated artificial neural network was trained, and unbalanced dataset and network tuning problems were faced. The obtained results improved the state-of-the-art in performing such kind of classification task.

In the future, we will investigate how to minimize the number of empirical assumptions in the feature extraction process and incorporate a weighted classification among the folds; additionally, a feature analysis will be performed to identify the best ones.

Moreover, novel techniques to face the dataset unbalancing problem and based on oversampling methods might be investigated [20]. It would also be interesting to evaluate deep learning approaches [21–24] and perform a direct comparison with the methodology proposed in this paper. A preliminary study regarding different deep learning semantic segmentation techniques applied to WSIs have been already conducted [25]. Finally, the presented workflow will be integrated into a complete CAD tool for kidney biopsies analysis.

## Methods

**Fig. 2:**
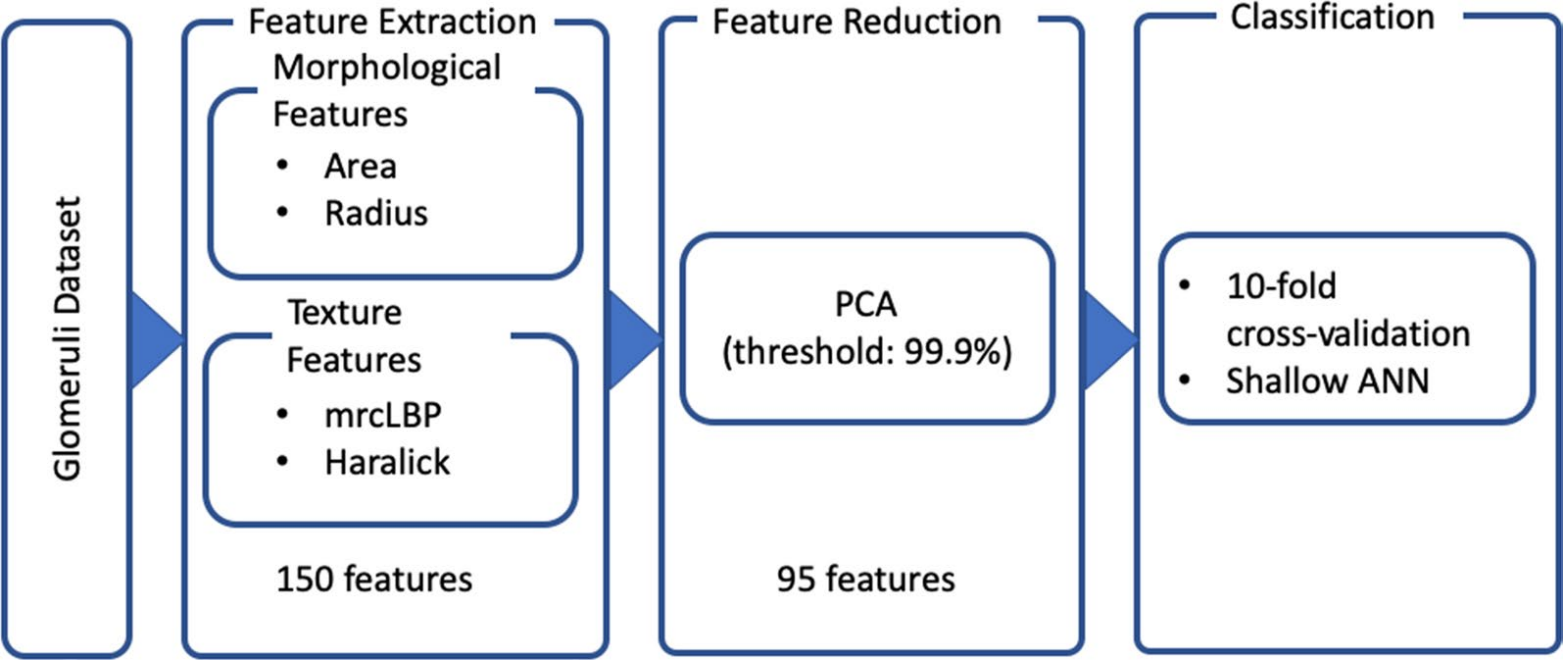
Full features extraction and classification work-flow

In this study, we present a CAD framework that allows the classification of the glomerulus condition using a feature-based approach. The proposed solution, that is based on image processing and machine learning techniques, has been designed to automatic label each glomerulus as sclerotic or non-sclerotic. A detailed representation of the full workflow for glomeruli classification is depicted in Fig. 2. The processing pipeline can be organized into three main steps: (i) feature extraction; (ii) feature reduction; (iii) classification.

### Data description

Whole Slide Images were collected between July 2011 and February 2015 by physicians from the Department of Emergency and Organ Transplantations of the Bari University Hospital (Italy). All the kidney biopsies with PAS staining were scanned by using the Aperio ScanScope CS at 20× with a resolution of 0.50 µm/pixel. The WSIs that have been considered within this study were collected from a total of 26 kidney digital biopsies of 19 donors and stored at full resolution in SVS file format (an Aperio file format consisting of pyramidal tiled TIFF with non-standard metadata and compression).

**Fig. 3:**
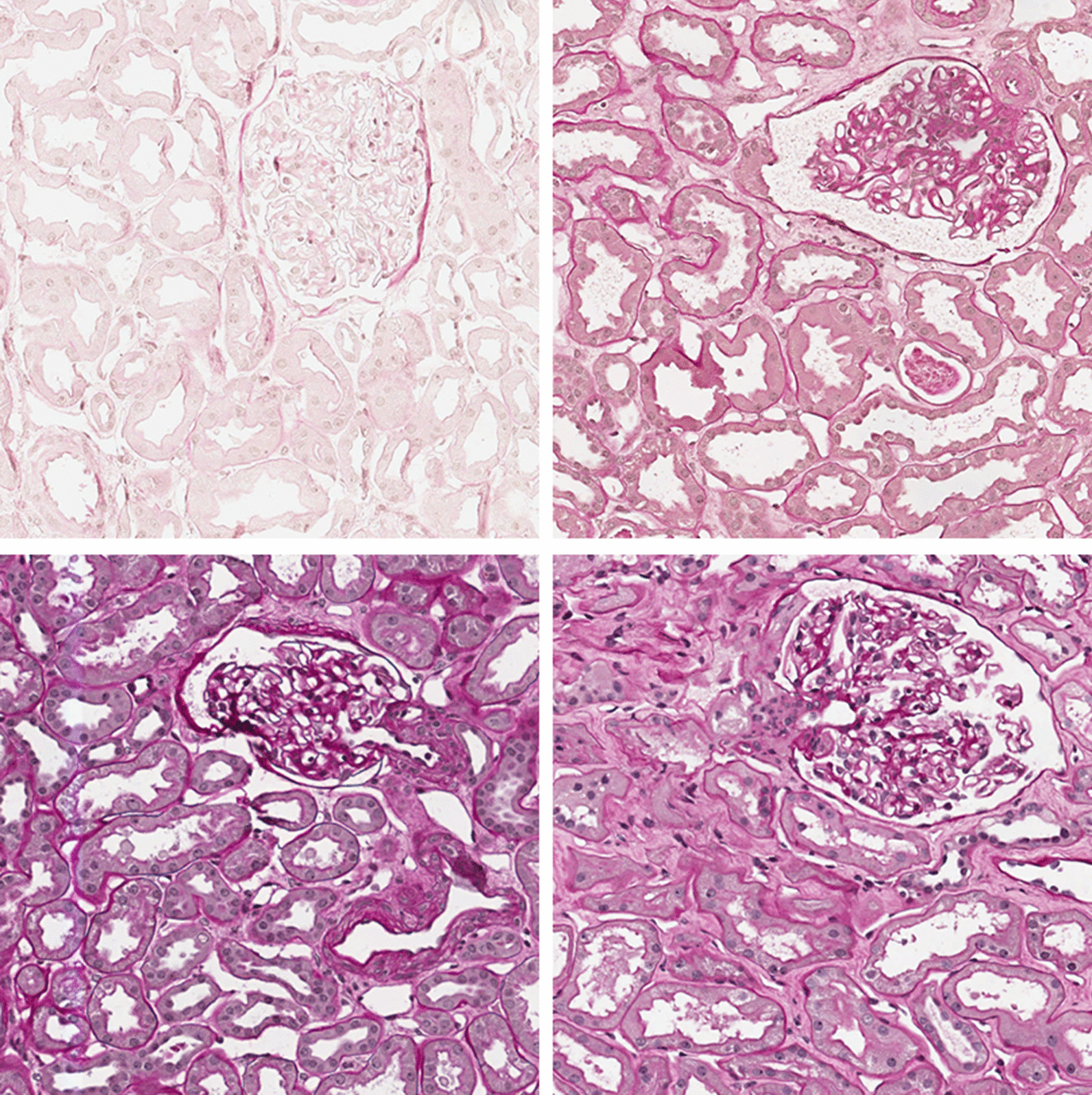
Examples of glomeruli with different saturations levels in PAS stain kidney biopsy

Each WSI contains a different number of biopsy sections (from one to seven). The whole used dataset counts an average of four biopsy sections per WSI and a total amount of 105 sections. The collected images of the used dataset are characterized by wide differences in terms of color and saturation, even if all of them have been treated with PAS staining. Examples of saturation differences are reported in Fig. 3.

### Dataset creation

All the glomeruli were manually identified and labelled by two medical graduands. Then, one expert renal pathologist validated the final annotations. The procedure consisted in outlining the real glomeruli region and labelling each glomerulus as sclerotic or non-sclerotic by using the Aperio ImageScope tool.

Due to the variability introduced with the manual annotation, each labelled region was surrounded by a rectangular bounding box with a 1.1 overestimation factor for each dimension. Then, all the detected and labelled glomeruli regions were extracted and used for creating the dataset.

The obtained initial dataset was composed of 428 sclerotic glomeruli and 2344 non-sclerotic glomeruli, with a ratio between the two classes of 1/5.5. In detail, a total of 2772 glomeruli were labelled and, on average, each biopsy and each section contained 106 and 26 glomeruli, respectively.

The dataset was subsequently divided into the train and test sets. In particular, the 20% of the original dataset has been used as test set, and the information of the test-set target has been used to assess final performances only. The selection has been randomly performed with the constraint that if a glomerulus appeared in the test-set, all the other glomeruli belonging to the same biopsy must appear in the test-set, meaning that the train/test division has been performed at biopsy level. The latest dataset configuration is reported in Table 1.

### Features extraction

The features extraction is the first step of the workflow that allows to define of a set of characteristics used to discriminate between the two different types of glomeruli. Based on the human reasoning used by the physicians able to address the problem, the best features to face the problem are those related to two main image processing techniques: morphological and texture-based features.

**Fig. 4:**
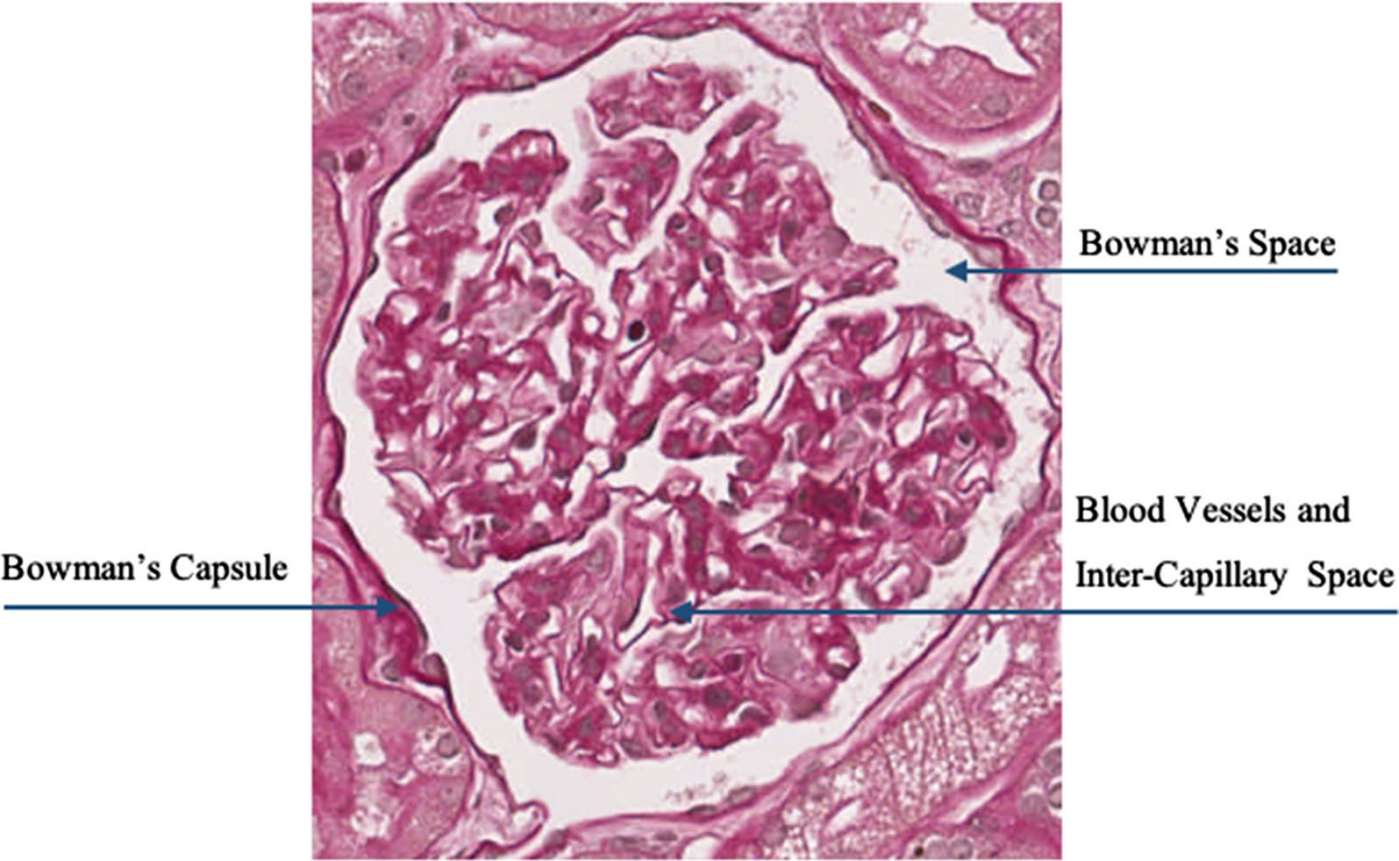
Main non-sclerotic glomerulus sections

As suggested by the pathologist involved in the study, the main differences between sclerotic and non-sclerotic glomeruli are about the shape of the Bowman’s capsule, the dimension and the texture due to blood vessels. Non-sclerotic glomeruli usually are characterized by an elliptic shape and the presence of the Bowman’s capsule that is separated from the capillary tuft with the mesangium by the Bowman’s space. The ensemble of the nuclei of cells (blue points in Fig. 4), the capillaries lumen (white areas in Fig. 4) and the mesangial matrix (regions with similar tonality and different levels of saturation in Fig. 4) show a particular texture commonly called “pomegranate texture”. Sclerotic glomeruli, instead, are characterized by an increase in the extracellular matrix that obliterates the capillaries lumen and by a reduced or absent Bowman’s space due to collagenous material.

**Fig. 5:**
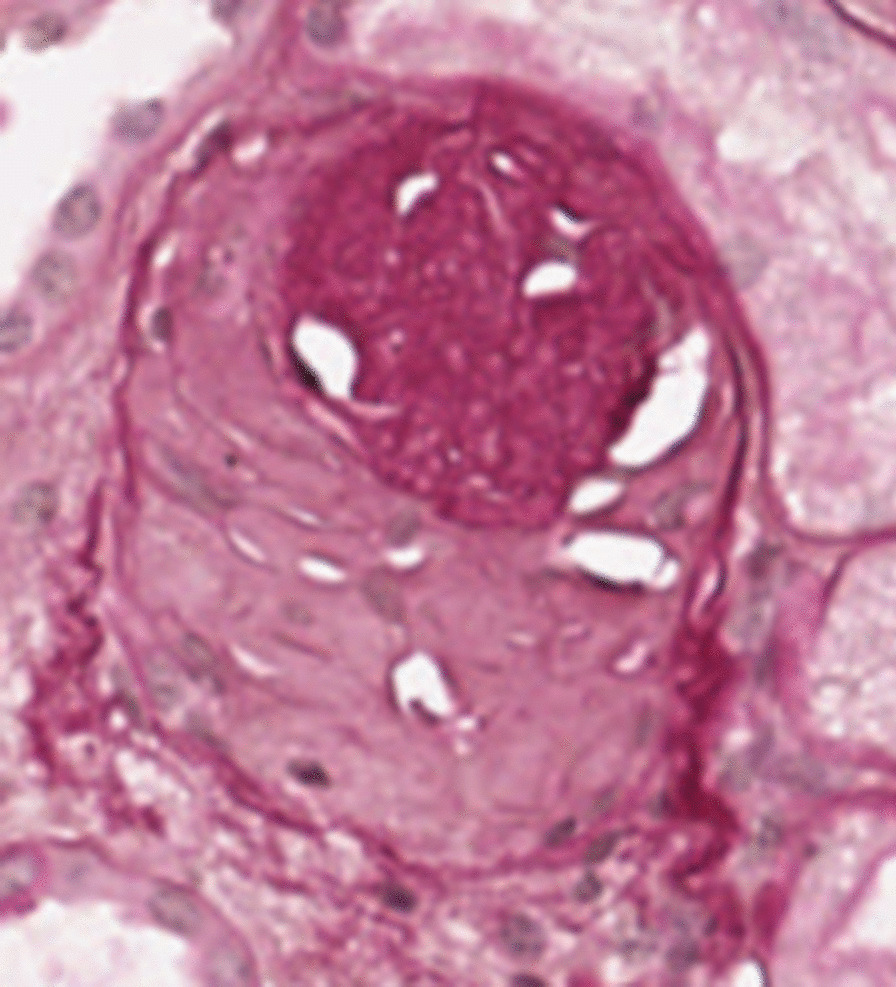
Example of sclerotic glomerulus

Figures 4 and 5 report an example of non-sclerotic and sclerotic glomerulus, respectively. It is worth noting that the fine-tuning of the image processing algorithms, including the parameter values and the algorithm configurations have been done on train set only.

#### Morphological features

Regarding the morphological features, we have considered two features that are related to the Bowman’s capsule and the Bowman’s space. The first feature is computed as the sum of the areas related to the Bowman’s capsule, the blood vessels areas and the inter-capillary spaces that are characterized by a whiteness coloration due to the PAS staining. The detection of the mask describing the region is based on three parallel image processing procedures that took into account the channels of three different color space: RGB, CMYK and Lab. In detail:

- the green channel of RGB colour space, since it is the most representative of the glomerulus structure;
- the complementary of magenta from the CMYK colour model has been chosen due to the detectable empirical significance of this colour component (Figs. 3, 4 and 5);
- *a* and *b* components of Lab colour space due to the link with the human colour vision.

**Fig. 6:**
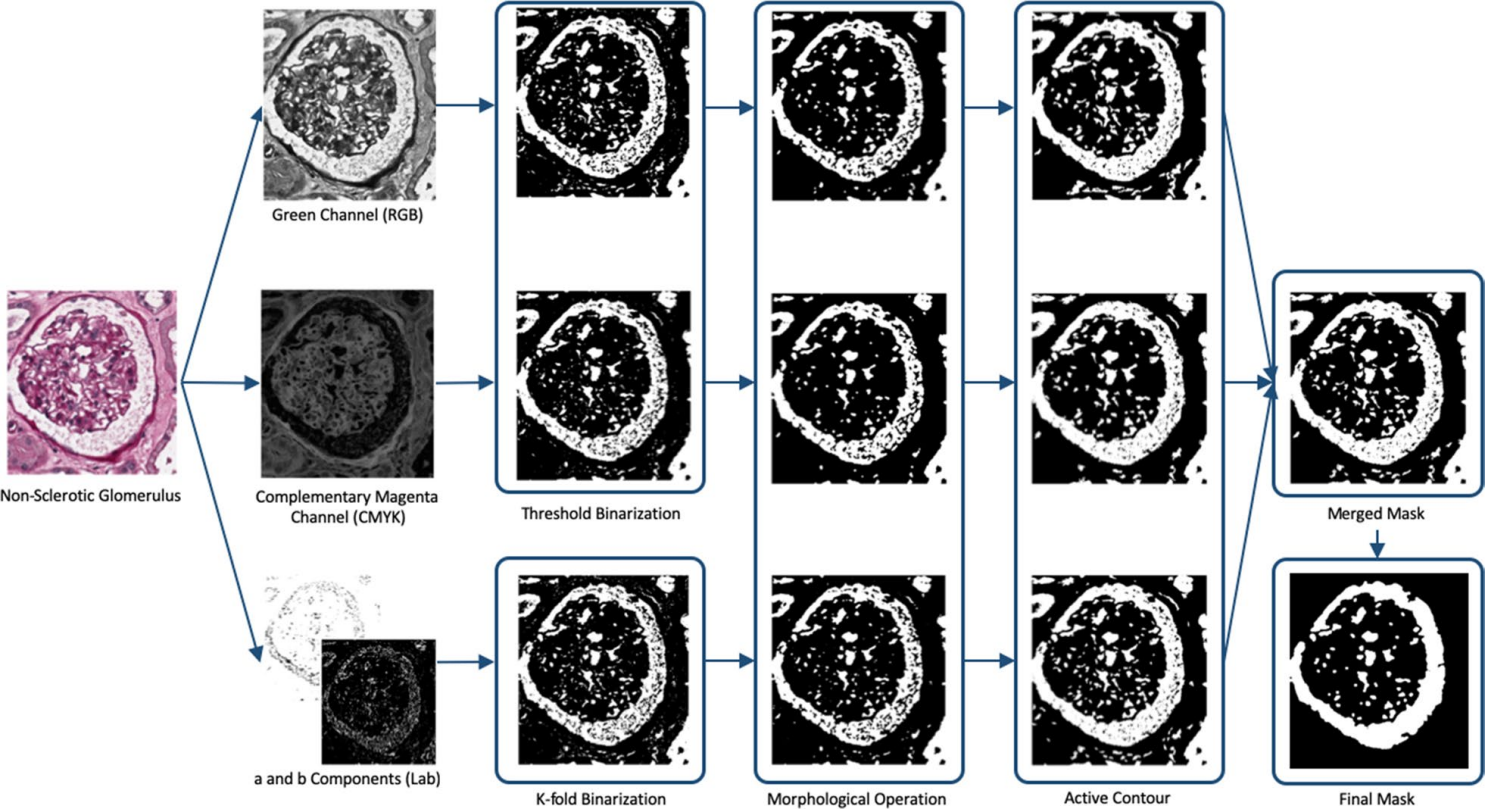
Example of application of morphological features work-flow on non-sclerotic glomerulus

**Fig. 7:**
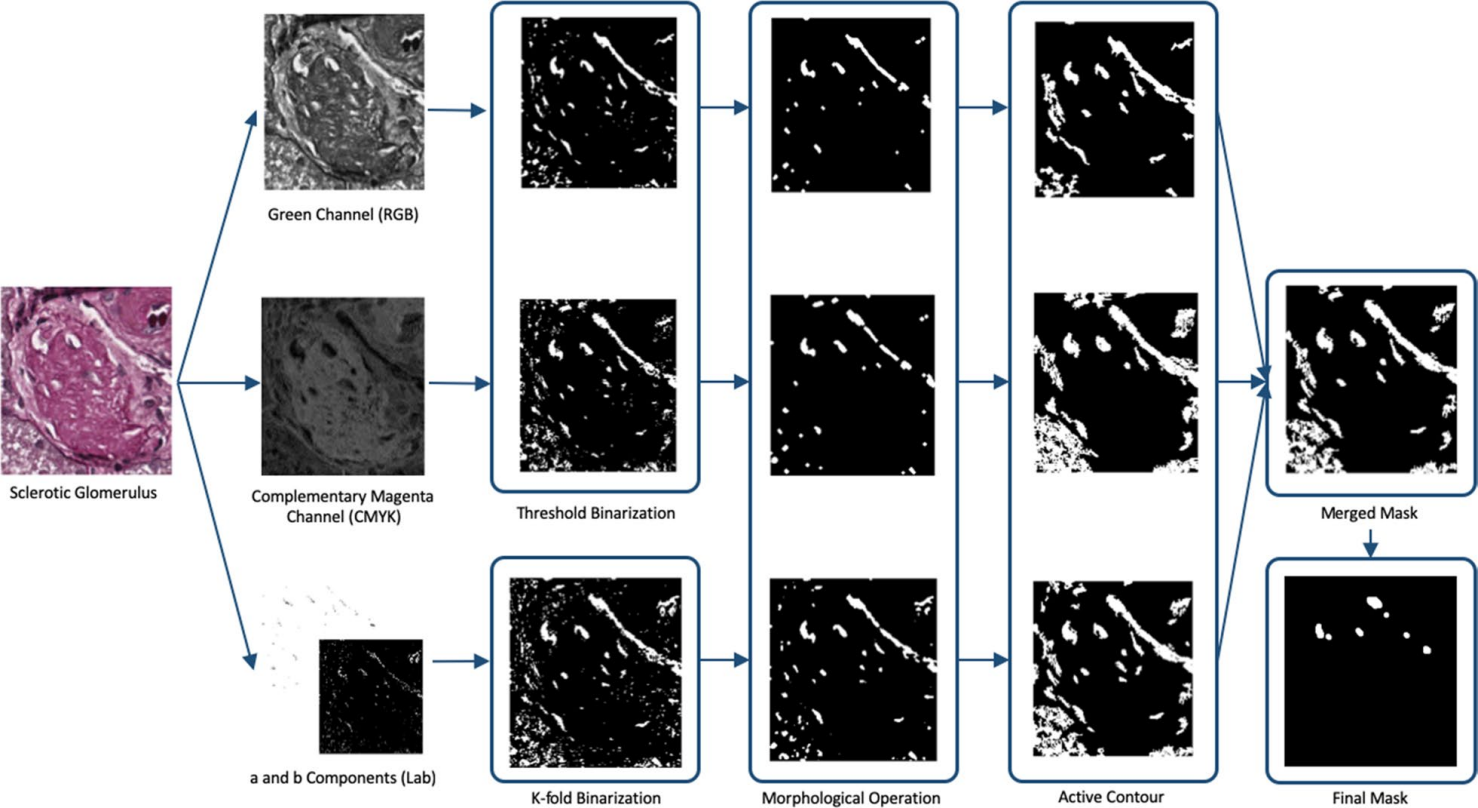
Example of application of morphological features work-flow on sclerotic glomerulus

An example of the application of the processes on non-sclerotic and sclerotic glomeruli is reported in Figs. 6 and 7, respectively.

The extraction of the masks for green channel from RGB colour space and for magenta channel from the CMYK colour model, follows the same image processing steps:

1. *Binarisation*: to keep the pixels related to white regions a threshold value has been empirically set to 190 [16];
2. *Morphological operators*: to clean the image obtained from the previous step, erosion, dilation and median filtering have been used with a disk of radius ranging from 1 to 3 as structuring element;
3. *Active contour*: to clean the shape of the obtained mask, active contour algorithm [26] has been used with 200 iterations (the chosen number of iterations avoid an extreme smoothing of the glomerulus shape).

The third mask was computed from *a* and *b* components of Lab colour space. The *ab* matrix has been used as input to k-means clustering algorithm [27]. In particular, the number of clusters was empirically set to 5, and the number of repetitions of the clustering process was set to 3 in order to set different initial cluster centroid positions for avoiding local minima. The mask was computed subsequently by retaining only those pixels belonging to the cluster with the greatest mean grey-scale intensity value. Then the steps 2 and 3 of the green-magenta segmentation process were applied.

**Fig. 8:**
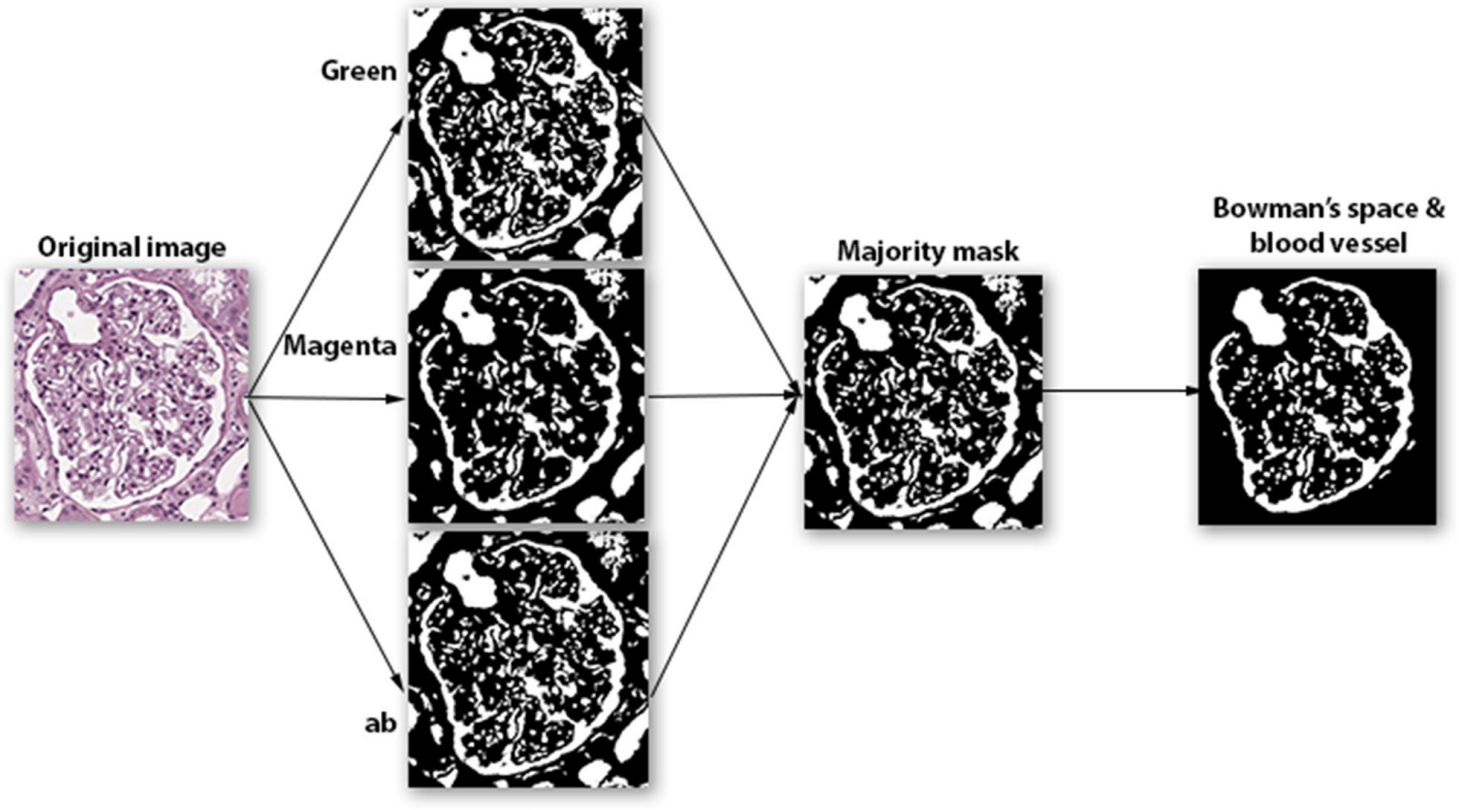
Work-flow of Bowman’s space segmentation

Finally, the three masks have been used to compute the final mask by using a majority criterion: only the pixels belonging to at least two masks were kept. The obtained mask was processed to remove artifacts and not interesting regions. In detail, too small regions (lesser than 1000 pixels), and a logical AND with a circle of radius equal to the smaller dimension of the image subtracted by 1/8 of its value was performed. Figure 8 shows the overview of the Bowman’s space segmentation workflow.

**Fig. 9:**
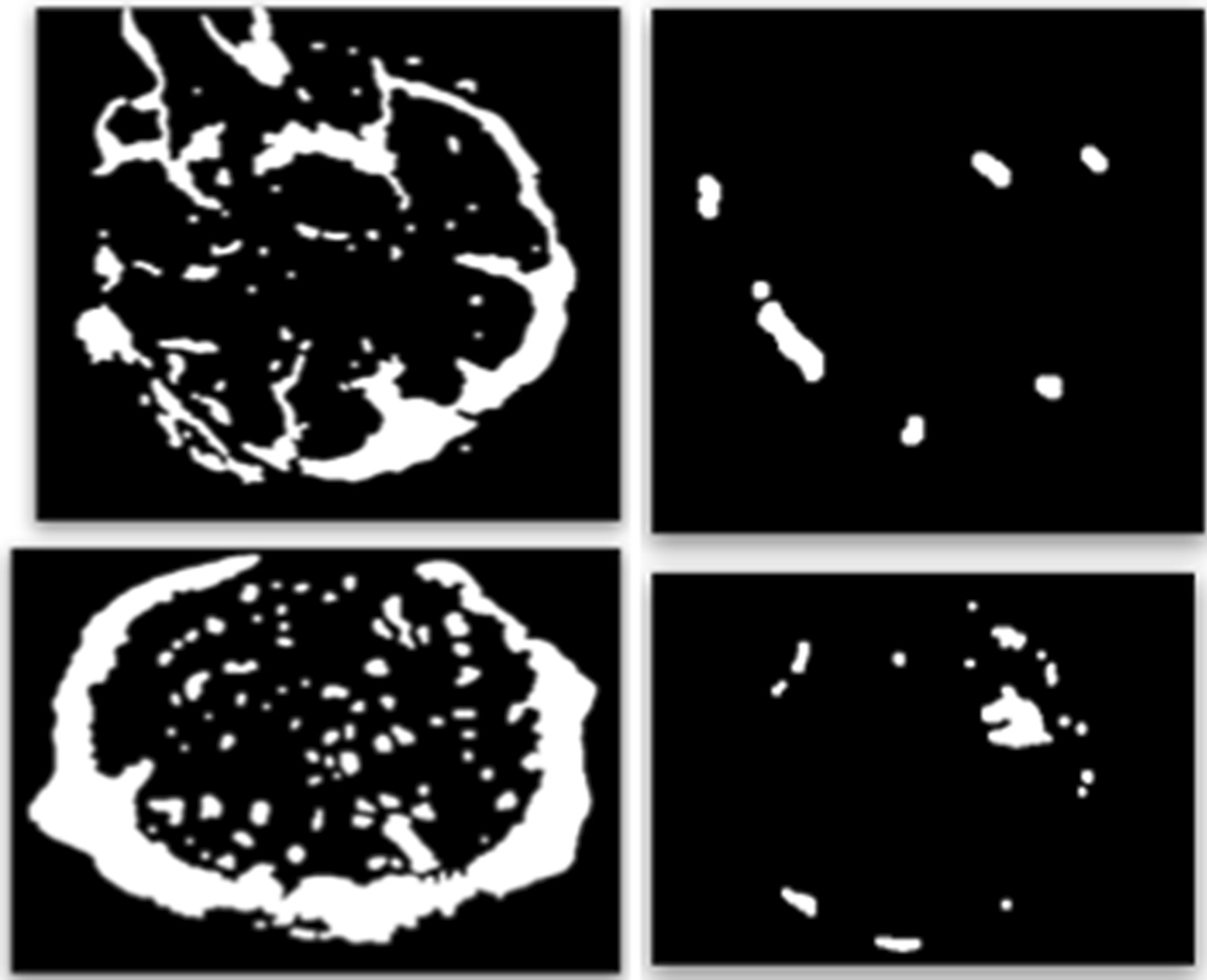
Results Comparison between the application of Bowman’s space segmentation on non-sclerotic (left) and sclerotic (right) glomeruli

Starting from the final mask (Figs. 6 and 7), the feature of interest was the sum of Bowman’s space, blood vessels and the inter-capillary region of the glomerulus, that is, in our workflow, the area corresponding to the white region. This value was finally normalized considering the image area. Figure 9 shows a comparison of the results of the workflow mentioned above applied on sclerotic and non-sclerotic glomeruli.

The second morphological feature considers the radius of the glomerulus. As first step, the convex hull containing all these regions was computed. Then, considering the convex hull ROI as a circle, the radius of a circle with the equivalent area was computed. As a result of the morphological workflow, a total of two features were computed: the area and the radius.

#### Texture features

Due to the particularity of the glomerulus texture and the differences in blood vessels and inter-capillary space between sclerotic and non-sclerotic, two well-known texture analysis algorithms were used: Local Binary Pattern (LBP) and Haralick features.

As already proposed in [13], multi-radial colour LBP (mrcLBP) is a suitable variation of classical LBP to face the glomerulus identification problem. In detail, it considers the application of the LBP algorithm to the three RGB colour channels with different radius values (1, 3, 9 and 27) and with invariance to rotation. Such configuration was applied to the raw RGB glomerulus images. The obtained features were ten for each radius, thus obtaining a total number of 120 features (10 features per radius, 4 radius, three channels).

The second set of texture-based features were based on the Haralick features. The four Grey-Level Co-occurrence Matrix, one for each direction, has been computed; then, the 14 Haralick indexes were computed, leading to 56 features. To reduce this number, the mean and the range among the four directions was then computed. Hence, the final number of features was 28 (14 mean and 14 range, one for each Haralick feature). As a result of the texture features extraction, a total of 148 features were computed.

### Features preprocessing

As described above, the feature extraction process generated 150 features that considered both the morphological and texture-based characteristics of the glomeruli. The Principal Component Analysis (PCA) was applied as feature reduction algorithm to reduce the correlation among the different features that will be used as inputs of the classification step. Before PCA, each feature was z-score normalized.

As stated before, the fine-tuning of the image processing and the classification algorithms has been conducted only on the train set. The feature reduction algorithm, instead, did not need or use the label information. For this reason, the application of PCA could be executed on the entire dataset or on the train dataset only, with different advantages and drawbacks. Both the solutions were applied on the dataset, and due to the complexity of the classification problem, 99.9% of variance has been chosen as the threshold value. Finally, 95 and 93 features where obtained when the PCA have been applied to the whole dataset and the train set only, respectively. Since the two approaches led to a similar number of features, we have chosen to take into account all the information inside the dataset, thus the number of features considered for the classification phase was 95.

### Glomeruli classification

The glomeruli classification steps are based on ANN and specifically on a shallow ANN architecture. The design of the ANN architecture and the tuning of its parameters were taken considering the train set only, whereas all the reported results and performance discussions refer to the test set (see Results and Discussion). K-fold (k was set to 10) was used as cross-validation technique to generalize, avoid overfitting and obtain a classifier independent from the specific input dataset. Several network initializations for each fold and hard voting among the folds were used both to obtain independency from a particular network initialization and to compute the overall fold class label.

The fixed training parameters were the following: one hidden layer, tansig and softmax as activation functions for the hidden and output layer, respectively; crossentropy as loss function; scaled conjugate gradient as backpropagation algorithm. A training early stop criterion, based on the validation set, was implemented to promote generalisation and to avoid overfitting; the stop criterion occurs if performance on validation set did not decrease inside a sliding window of 6 epochs.

**Fig. 10:**
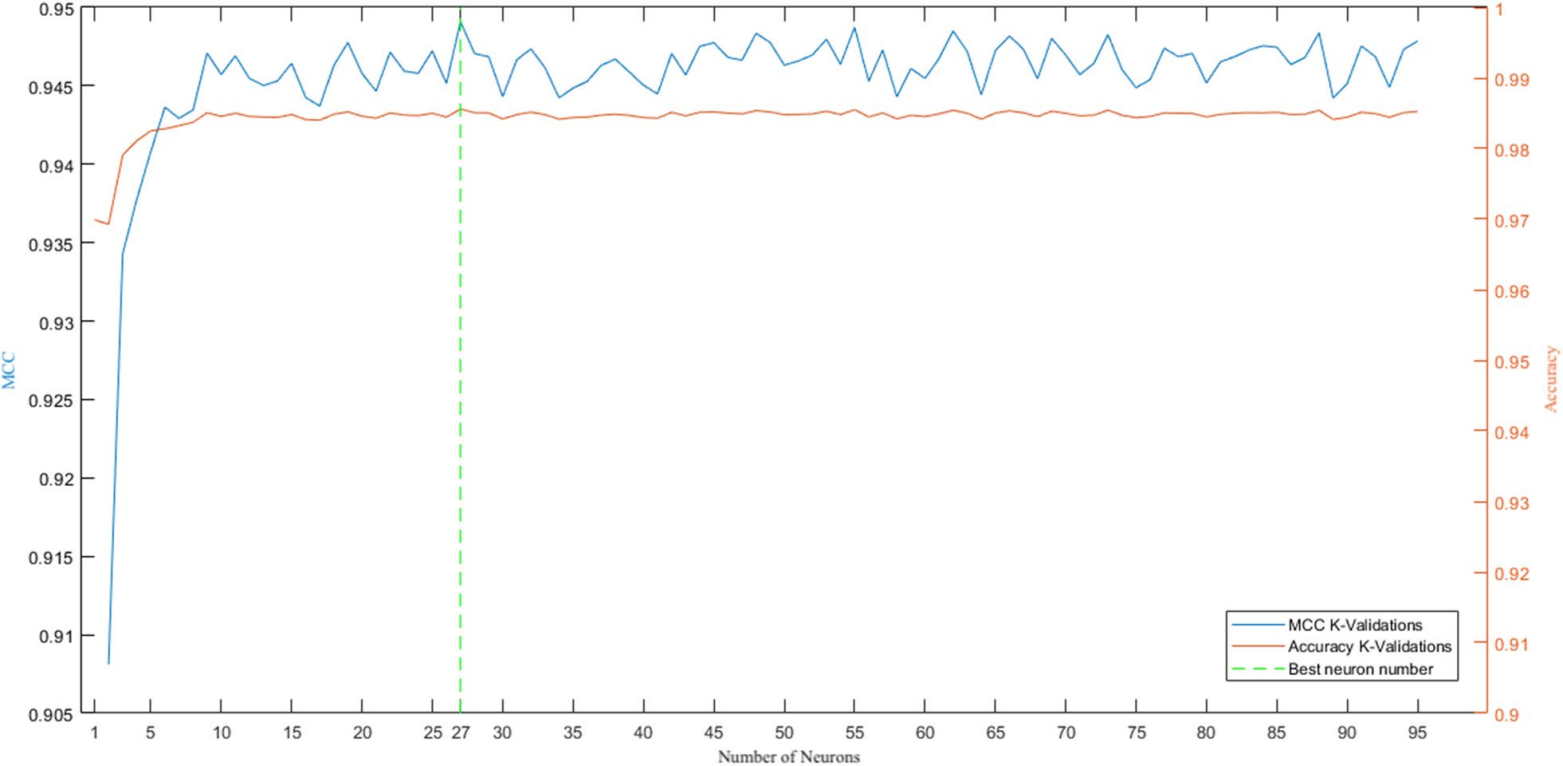
MCC and accuracy trend based on number of neurons

The number of neurons of the hidden layer has been selected as follow. The performance of 95 networks were compared. In detail, several networks with the hidden layer size ranging from 1 to 95 were trained (it worth remembering that 95 is the number of the input features). Among the 95 evaluated topologies, the one with a hidden layer size equal to 27 has been selected based on the best MCC value computed as the mean MCC of the folds. A graphical representation of the trend of MCC and accuracy indexes is shown in Fig. 10; the final Artificial Neural Network configuration is summarized in Table 6.

**Table 6:** Artificial neural network configuration

Parameter name	Value
# input	95
Topology	[27*, 1]
Activation functions	[tansig, softmax]
Loss function	Cross-entropy
Backpropagation algorithm	Scaled conjugate gradient
Early stop criterion	Validation fail*
Cross-validation method	k-fold (k = 10)

*An in-depth explanation about the neurons number choice and early stop criterion is reported in Section *Glomeruli Classification*

#### Unbalanced dataset problem

As reported above, the training set was affected by a heavy unbalanced distribution between sclerotic and non-sclerotic glomeruli (5.5 non-sclerotic glomeruli for each sclerotic glomerulus). In order to avoid overfitting in the training phase, data augmentation was not considered a suitable solution since the selected features are invariant to the main image transformations. Hence, we considered the following approach.

Firstly, we have considered the use of the MCC as a general performance comparison among the folds. As reported in Eq. 4, MCC takes into account false negatives and false positives, and computes a correlation coefficient between predicted and target classes. This coefficient can range within the interval [− 1; 1], where 1 indicates perfect prediction, − 1 complete disagreement and 0 is equivalent to the random predictor. As stated in [28], among the usual performance scores, MCC is the only one that takes into account the ratio of the confusion matrix size, and it revealed to be a better index of performance than accuracy or F1 score on unbalanced datasets.

Concerning the selection of the correct classification threshold value, the Receiving Operating Characteristic (ROC) curve has been used. Two approaches were analyzed. The first one (Approach A) assumes the optimal value as the first intersection point between the ROC curve and a line with slope equal to the ratio between the total number of negative and positive samples and sliding from the upper left corner of the ROC plot ((FPR, TPR) = (0, 1)). Whereas, the second approach (Approach B) [29] evaluates the point of minimum distance (see Eq. 5) from the point (0, 1) of the ROC plot.

The comparison of the two methods (Approach A and Approach B) in terms of different performance indexes (Eq. 1, 2, 3 and 4) is reported in Table 7. Since in the medical domain, a correct prediction of positives to a disease is more important than the prediction of negatives, a higher recall is preferred, thus the Approach B was chosen.

5 $$\mathop {\min }\limits_{i} \sqrt[2]{{\left( {1 - sensitivity\left( i \right)} \right)^{2} + \left( {1 - specificity\left( i \right)} \right)^{2} }}$$

**Table 7:** Comparison between the two ROC thresholding approaches

	Approach A	Approach B
Accuracy	0.9898	0.9865
Precision	0.9775	0.9332
Recall	0.9575	0.9880
MCC	0.9612	0.9520

The reported values are the mean among the ten-fold

## Data Availability

The datasets generated and/or analysed during the current study are not publicly available due privacy concerns, but are available from the corresponding author on reasonable request.

## Abbreviations

ANN: Artificial neural networks
CAD: Computer aided diagnosis
CKD: Chronic kidney disease
ECD: Expanded criteria donor
H&E: Haematoxylin and eosin
HOG: Histogram of oriented gradients
LBP: Local binary pattern
MCC: Matthews correlation coefficient
mrcLBP: Multi-radial colour LBP
PAS: Periodic acid–Schiff
PCA: Principal component analysis
ROC: Receiving operating characteristic
ROI: Regions of interest
SVM: Support vector machine
WSI: Whole-slide image
